# Reduced Cross-Frequency Coupling and Daytime Sleepiness in Obstructive Sleep Apnea Patients

**DOI:** 10.3390/biology11050700

**Published:** 2022-05-02

**Authors:** Haralampos Gouveris, Nabin Koirala, Abdul Rauf Anwar, Hao Ding, Katharina Ludwig, Tilman Huppertz, Christoph Matthias, Sergiu Groppa, Muthuraman Muthuraman

**Affiliations:** 1 Sleep Medicine Center, Department of Otolaryngology, University Medical Center, Johannes Gutenberg University Mainz, 55131 Mainz, Germany; katharina.ludwig@unimedizin-mainz.de (K.L.); tilman.huppertz@unimedizin-mainz.de (T.H.); christoph.matthias@unimedizin-mainz.de (C.M.); 2 Haskins Laboratories, Yale University, New Haven, CT 06511, USA; nabin.koirala@yale.edu; 3 Department of Biomedical Engineering, University of Engineering and Technology (New Campus), Lahore 54890, Pakistan; raufanwar@uet.edu.pk; 4 Movement Disorders and Neurostimulation, Biomedical Statistics and Multimodal Signal Processing Unit, Department of Neurology, University Medical Center of Johannes Gutenberg University Mainz, 55131 Mainz, Germany; kenhding@uni-mainz.de (H.D.); segroppa@uni-mainz.de (S.G.); mmuthura@uni-mainz.de (M.M.)

**Keywords:** obstructive sleep apnea, EEG, cross-frequency coupling, polysomnography

## Abstract

**Simple Summary:**

Obstructive sleep apnea (OSA) is one of the most common breathing-related sleep disorders. The processes that take place in specific areas of the brain during distinct sleep stages and specific respiratory events are suspected to be impaired in OSA patients. These brain processes may be described in complex (mathematical) terms. Using one of these descriptions (phase–amplitude cross-frequency coupling), we demonstrated in electroencephalographic (EEG) recordings of OSA patients that the process sequences in certain areas of the brain are altered in distinct sleep stages of OSA patients compared to patients who do not suffer from clinically relevant OSA. In addition, we were able to find a physiological marker that assesses the severity of daytime sleepiness, one of the main symptoms of OSA. This study supports the hypothesis that OSA is a neuronal/neuromuscular disorder. Thus, it contributes to a better understanding of the so-far-unexplained cause for the development of OSA and opens new possibilities for the exploration of alternative therapeutic approaches.

**Abstract:**

Obstructive sleep apnea (OSA) is associated with sleep-stage- and respiratory-event-specific sensorimotor cortico-muscular disconnection. The modulation of phase–amplitude cross-frequency coupling (PACFC) may influence information processing throughout the brain. We investigated whether sleep-stage-specific PACFC is impaired at the sensorimotor areas in OSA patients. C3 and C4 electrode EEG polysomnography recordings of 170 participants were evaluated. Different frequency band combinations were used to compute CFC modulation index (MI) to assess if MI differs between OSA and non-significant OSA patients in distinct sleep stages. We tested if the CFC-MI could predict daytime sleepiness in OSA. Theta–gamma CFC-MI at cortical sensorimotor areas was significantly reduced during all sleep stages; the delta–alpha CFC-MI was significantly reduced during REM and N1 while increasing during N2 in patients with respiratory disturbance index (RDI) > 15/h compared to those with RDI ≤ 15/h. A sleep stage classification using MI values was achieved in both patient groups. Theta–gamma MI during N2 and N3 could predict RDI and Epworth Sleepiness Scale, while delta–alpha MI during REM predicted RDI. This increase in disconnection at the cortical sensorimotor areas with increasing respiratory distress during sleep supports a cortical motor dysfunction in OSA patients. The MI provides an objective marker to quantify subjective sleepiness and respiratory distress in OSA.

## 1. Introduction

Obstructive sleep apnea (OSA) is highly prevalent in adults [1]. The disorder may cause neural injury, or OSA itself may be a neurologic disorder [2,3]. The stimulation of the hypoglossal nerve serving as an effective treatment for many OSA patients supports the argument for OSA being a neuronal/neuromuscular disorder [4]. Accordingly, evidence of a disconnection between motor-associated cortical neural groups and peripheral upper cervical motor units was found in OSA [5]. Additionally, there is evidence from functional MRI studies for a disconnection between the sensorimotor and other cortical regions in OSA [6,7].

Cross-frequency coupling (CFC) is a fundamental feature of brain oscillatory activity and is highly correlated with brain function [8]. CFC encompasses various patterns: phase synchronization, amplitude co-modulation and phase–amplitude coupling (PAC). In the present study, we investigated PAC, as it represents neural coding and information transfer within local microscale and macroscale neural ensembles of the brain [9,10]. Low-frequency oscillatory activity depicts the regulation of information flow among the brain areas by modulating the excitability of local brain ensembles [11]. The phase of this low-frequency oscillatory activity affects both the rhythm of high-frequency activity and the rate of firing of individual neurons [10]. Therefore, phase–amplitude cross-frequency coupling (PACFC) seems to promote an effective interaction between neurons of similar phase preferences and the synchronization of high-frequency bands during specific slower rhythm phases [12].

Understanding the neurophysiological connectivity patterns and oscillatory dynamics at the sensorimotor cortical areas may help to further elucidate the pathophysiological central nervous system (CNS) processes in OSA. Additionally, given that OSA is clinically associated with cognitive, vigilance and alertness disorders in the awake state (especially, excessive daytime sleepiness) [13,14], better understanding and modelling of such connectivity may lay the ground to predict relevant clinical phenomena.

Sleepiness, a common patient-reported outcome in OSA, exhibits significant associations with functional connectivity within the sensorimotor network [15]. A functional neuroimaging study in adults older than 55 years showed hypoperfusion in sensorimotor areas of patients with severe OSA [16]. Further, neuroimaging studies of interhemispheric interaction after sleep deprivation have strongly associated sleepiness with activity of the bilateral precentral gyrus, an important part of the sensorimotor network [17]. The study of CNS-associated disorders in OSA (including OSA pathogenesis itself) and the mode of action of CNS-pharmacological approaches on sleep-related disorders will benefit from such targeted combined neurophysiologic/neuroimaging study approaches.

In this study, we aimed to characterize the functional network connectivity of the sensorimotor area in therapy-naive OSA patients. For this, we initially tested if sleep-stage-specific theta–gamma PACFC modulation differs between patients with and without significant OSA. This assessment was based on the findings from previous studies, which have shown a significant role of theta oscillation during different sleep stages [18]. Moreover, we also investigated if these modulations were specific to a particular sleep stage. To assess whether any possible functional disconnection at the sensorimotor area was frequency specific, as a control experiment, we further compared delta–alpha PACFC modulation between the patients with and without significant OSA. Finally, we examined if such a disconnection was correlated to clinical parameters, such as patient-reported sleepiness outcomes (Epworth Sleepiness Scale; ESS).

Previous studies have used the cross-frequency coupling for sleep stage classification primarily in healthy adults but also in OSA patients [19,20,21]. However, none of these studies assess the importance of these classifications in terms of clinical transferability for OSA patients. In this study, we use the frequency-specific CFC-based modulation indices not only for sleep stage classification but to further show that the same modulation indices could also predict clinical scores, such as ESS. Hence, these findings should help us unravel whether either a frequency–specific, sleep-stage-specific or global functional disconnection exist(s) in the sensorimotor area of OSA patients. Additionally, this might provide an objective neurophysiologic surrogate marker to quantify patient-reported subjective sleepiness as well as the severity of the respiratory disease in OSA patients.

## 2. Methods

### 2.1. Study Participants

A total of 170 participants were included in the study. The patients were divided into the main and validation dataset, with 86 participants in the main dataset: 22 females, age range: 27–84 years, 44 subjects with moderate or severe OSA; and 84 in the validation dataset: 28 females, age range 35–75 years, 42 subjects with moderate or severe OSA. Data used for the analysis were assessed retrospectively. Consecutive datasets of patients after application of the aforementioned inclusion and exclusion criteria were included for analysis. Therefore, a retrospective unrandomized case–control study design was used. All patients presented here had first visited the outpatient department of the sleep medicine center of an academic tertiary medical center because of complaints of snoring and/or daytime sleepiness. In all participants, the diagnosis of OSA was first established using the PSG recordings used in this study. Hence, all participants were therapy naive with no positive airway pressure therapy, upper airway surgery or therapy with any mandibular advancement devices having been previously used. The study protocol was approved by the local institutional review board (Nr. 2018-13942).

Inclusion and exclusion criteria for participants were based on conditions that may have influenced the emergence and degree of observed OSA and/or the EEG recordings. Included were the data of adult patients (aged > 18 years) who presented with complaints of snoring and/or daytime sleepiness in the outpatient department, had no previous therapy for sleep-disordered breathing and underwent full-night polysomnography in our sleep medicine center. Participants with neurodegenerative (e.g., Parkinson’s disease) or neuro-inflammatory (e.g., multiple sclerosis) disorders, history of stroke, heart failure based on New York Heart Association (NYHA)—stage 3 or 4, chronic obstructive pulmonary disease (COPD), and any psychiatric disorders were excluded from the study. In addition, those with a history of regular use of sedatives, benzodiazepines, serotonin-uptake inhibitors or other psychotropic medication, malignant disease of any kind, radiation therapy of any cranial or neck anatomic region, surgery on intracranial structures or surgery for therapy of sleep-disordered breathing (either snoring or OSA) were further excluded from the study. Patient-reported outcomes on excessive daytime sleepiness (EDS) were acquired using the Epworth Sleepiness Scale (ESS) [22,23].

### 2.2. Data Acquisition

Full-night polysomnography (PSG) recordings of 170 thoroughly tested individuals in the Sleep Medicine Center of Johannes Gutenberg University hospital who fulfilled the study entry criteria were used for analysis. All participants underwent overnight polysomnography (PSG) with recordings of electroencephalogram, electro-oculogram, a submental and pretibial electromyogram, and electrocardiogram. Polysomnography (PSG) was recorded according to current American Academy Sleep Medicine, Inc., Illinois, USA (AASM) standards to determine the nature and degree of sleep-disordered breathing. Nasal airflow was depicted by measurement of impact pressure through a nasal sensor in which pressure fluctuations of the breathed air stream were determined. Thoracic and abdominal excursions, oxyhemoglobin saturation (pulse oximeter) and body position were simultaneously recorded. Snoring was recorded with a pre-laryngeally fixed microphone. PSG recordings were performed with the Alice-LE-Diagnostic Sleep System (Philips Healthcare/Respironics, Best, Netherlands) for all patients. All EEG recordings were from C3 and C4 electrodes at the sampling rate of 200 Hz. The patients were divided into the main and validation dataset, with 86 participants in the main dataset: 22 females, age range: 27–84 years, 44 subjects with moderate or severe OSA; and 84 in the validation dataset: 28 females, age range 35–75 years, 42 subjects with moderate or severe OSA. The recording parameters (EEG band pass filter (0.05–200) Hz, sampling rate and EEG channels C2-M1 (left mastoid) and C4-M2 (right mastoid)) were identical in both datasets. All polysomnography (PSG) recordings were performed in a standardized setting between 10 p.m. and 6 a.m. for each patient.

### 2.3. Data Analysis (Preprocessing)

Sleep stages were visually (manually) scored according to the American Academy of Sleep Medicine, Inc. (Darien, IL, USA) guidelines [24,25]. Sleep-related respiratory events were visually (manually) scored according to the American Academy of Sleep Medicine updated guidelines [26]. Apnea was scored when there was a drop in the peak signal excursion by ≥90% of pre-event baseline for ≥10 s. Similarly, hypopnea was scored when the peak signal excursions dropped by ≥30% of pre-event baseline for ≥10 s in association with either ≥3% arterial oxygen desaturation or a cortical arousal. Further classification in obstructive, central or mixed respiratory apnea events was based on simultaneous evaluation of nasal airflow and thoracic and abdominal excursion.

**Figure 1:**
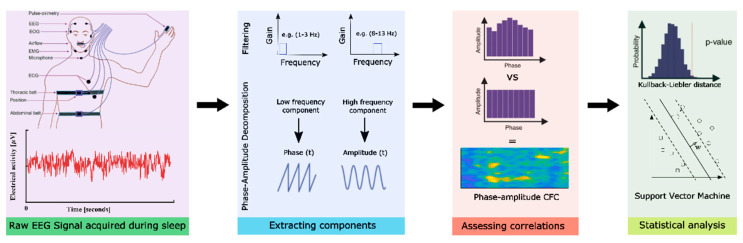
*Data acquisition and analysis pipeline overview*. Here, the first block represents the data acquisition, second block-EEG data pre-processing, third-computing modulation indices and the fourth block represents the statistical analysis performed.

The obtained EEG data preprocessing was performed using MATLAB (version 2019a, Mathworks Inc., Natick, Massachusetts, USA) and the fieldtrip toolbox [27]. For preprocessing, EEG raw data were low-pass filtered (fourth-order Butterworth filter; cut-off frequency: 100 Hz) to avoid aliasing, followed by high-pass filtering at 0.5 Hz [28]. For artifact removal, the data were subjected to independent component analyses (FastICA) to remove artifactual components related to muscle, eye blink, eye movement and line noise artifacts. The preprocessed data were then split into six different frequency bands, namely very low frequency (VLF, 0.1–1 Hz), delta (1–3 Hz), theta (4–7 Hz), alpha (8–13 Hz), beta (14–30 Hz) and gamma (31–100 Hz) for both electrodes. An overview of data acquisition and analysis pipeline is illustrated in Figure 1.

### 2.4. Data analysis (Modulation Index)

The phase to amplitude cross-frequency coupling (PACFC) indicates the modulation of high-frequency power by the low-frequency phase [29,30]. This CFC modulation index (MI) will then be used to identify the phase–amplitude association between the phase-modulating frequency band (for, e.g., delta) and amplitude-modulated frequency bands (for, e.g., alpha). To compute the CFC-MI, the following steps were carried out. First, the obtained EEG signal was filtered into two frequency ranges, namely, delta and alpha. After filtering, Hilbert transform was applied on both the filtered time series to obtain the phase of one time series and the amplitude envelope of the other. This combined time series will then have the information at each phase of delta oscillations to amplitude of alpha rhythm [31]. The possible phase range of −180° to +180° was then divided into 20 bins (N), each comprising 18°, and the Kullback–Leibler (KL) distance was calculated using the formula:

$$D_{KL}\left(P,Q\right)=\overset{N}{\underset{j=1}{\sum }}P\left(j\right)log\left[\frac{P\left(j\right)}{Q\left(j\right)}\right]$$

where *D* is the KL distance of a discrete distribution *P* from a distribution *Q* [32]. The KL distance has the property of being always greater than zero, i.e., *D_KL_ (P,Q)* ≥ 0, except when *P* and *Q* distributions are the same, i.e., *D_KL_ (P,Q)* = 0 *iff P* = *Q* and resembles the definition of Shannon entropy given as

$$H\left(P\right)=-\overset{N}{\underset{j=1}{\sum }}P\left(j\right)log\left[P\left(j\right)\right]$$

Hence, relating it to Shannon entropy, KL distance can be used to measures the divergence between the distribution of the data and the uniform distribution (*U*) as

$$D_{KL}\left(P,U\right)=log\left(N\right)-H\left(P\right)$$

Finally, the CFC-MI was calculated on the bins. The distribution of mean amplitude was uniform over all the bins, indicating no association between phase and amplitude [31,32,33]. So, the modulation index (*MI*) could be computed as

$$MI=\frac{D_{KL}\left(P,U\right)}{log\left(N\right)}$$

Therefore, if the mean amplitude is uniformly distributed over the phases, the *MI* would be zero, and it would be maximum if we obtain dirac-delta in the distribution of the phases. The coupling for theta–gamma and delta–alpha frequency bands was estimated between the amplitude of the higher-frequency signals and the phase of low-frequency signals by correlation. The CFC was estimated with a window length of 5 s with a 50% overlap [34].

### 2.5. Data Analysis (Statistical Analysis)

To investigate the significance of these CFC modulation indices, we used a support vector machine (SVM) algorithm to classify the different sleep stages based on CFC-MI values from both frequency bands. SVM is a powerful tool for non-linear classification between two datasets, which looks for an optimally separating threshold between the two datasets by maximizing the margin between the classes’ closest points [35]. Here, we used the polynomial function kernel for this projection due to its good performance, as discussed in Ref [35], and used the grid search (min = 1; max = 10) to find the few optimal input parameters and gamma (0.25). The selection was checked by 10-fold cross-validation by taking 75% of the data for training and 25% for testing. Moreover, to validate the effectiveness of these CFC modulations indices for clinical usage, we further applied SVM analysis to predict the clinical scores (RDI and ESS) used in the diagnostic criteria for OSA patients. Here, we performed a support vector regressor (SVR) analysis—representing a machine-learning-based multiple-regression method—that could associate the observed and trained values and present the prediction accuracy [36]. To obtain the threshold for prediction accuracy, we devised an approach based on the statistical inference obtained from the Bayesian credible interval [37]. The 75% threshold could differentiate the posterior distribution from the 95% Bayesian credible interval (indicating the inclusion of 95% of the data points). Here, the posterior distribution and the credible interval were obtained considering all modulation indices from all the sleep stages from both groups and the highest density interval containing 95% (range: 0.32–0.89) of the distribution. Hence, the prediction accuracy of the above (75%) obtained after 10-fold cross-validation was considered a fairly significant result.

### 2.6. Scientific Control

In order to assure that there are no effects of other variables other than the independent variable in the findings of the study, we conducted the following scientific control. (1) We validated whether the estimated PACFC is independent of the arousals and periodic limb movements from these patients. For this, we estimated the arousal index and periodic leg movement (PLM) index for each patient, and the Pearson correlation was estimated between the PACFC at each sleep stage and these two indices. (2) As previous studies have shown significant association between heart rate variability and OSA clinical scores [38,39,40,41], we validated whether the PACFC is influenced by the autonomic nervous system activity. For this, we estimated the heart rate variability (HRV) for each patient separately and estimated the Pearson correlation with the PACFC at each sleep stage. HRV was computed using the standard deviation of normal-to-normal intervals; a technique detailed elsewhere [42]. (3) We computed the power of the sample size used in the study. For this, we estimated the post hoc Bayesian posterior distribution analyses [43] for the MI index of the N1 sleep stage between the two groups.

### 2.7. Data Availability

The methodological tools software will be made available upon request to the corresponding author. The patients’ data used in the manuscript could be made available to a research institute in Germany given the appropriate ethics and research approval. The data could not be made available to an institute outside of Germany because of the consent and data agreement signed with the patients.

## 3. Results

### 3.1. First (Main) Dataset

From the 86 patients analyzed, 42 patients were diagnosed with a respiratory disturbance index (RDI) ≤ 15 per hour (4 with RDI < 5 per hour and 38 with RDI between 5 and 15 per hour), and 44 patients were diagnosed with clinically significant OSA (30 patients with RDI between 15 and 30 per hour and 14 patients with RDI > 30 per hour). These two groups did not significantly differ in terms of age and sex (*p* > 0.05). The demographic details along with clinical measures obtained are presented in Table 1. The statistical analysis performed for cross-frequency coupling (CFC) parameters and its association with clinical measures obtained from these two groups yielded significant results as detailed below.

**Figure 2:**
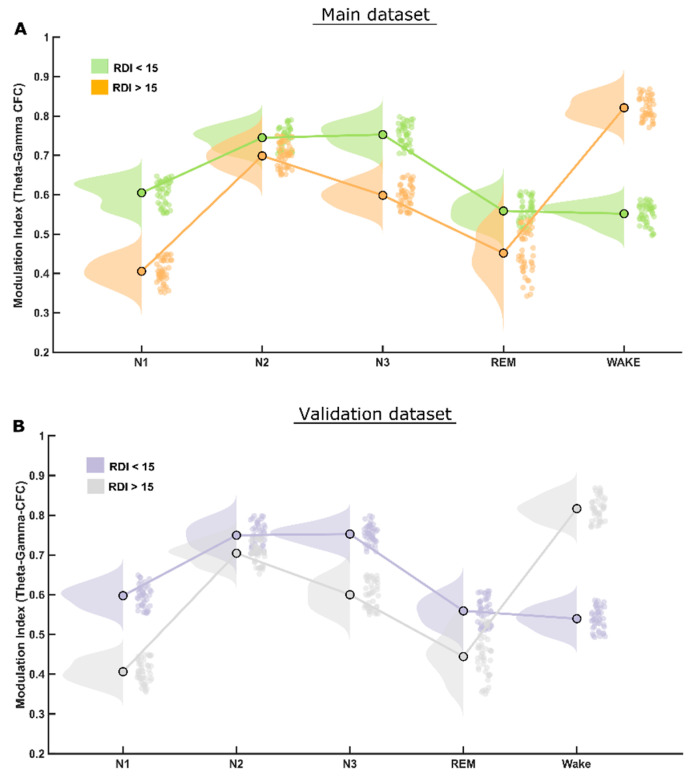
*MI differences in theta–gamma CFC*. (**A**) The raincloud plot clearly illustrates that patients of the RDI > 15/h group have significantly lower theta–gamma CFC modulation index than that of RDI ≤ 15/h group in all (NREM and REM) sleep stages. (**B**) Exactly the same pattern was found in both the initial and the validation patient groups.

The CFC modulation indices (MI) at the theta–gamma frequency bands were reduced significantly (*p* < 0.001) at all sleep stages in patients with clinically significant, i.e., moderate or severe, OSA (RDI > 15/h), as depicted in Figure 2A. The theta–gamma modulation index was higher during NREM stages N2 and N3 compared to N1 and REM sleep stages for both groups. The difference in the MI values between the two groups was highest during N1; it was reduced during N2 but increased again during N3 and REM sleep stages. A table depicting all these values is provided as Supplementary Table S1.

**Figure 3:**
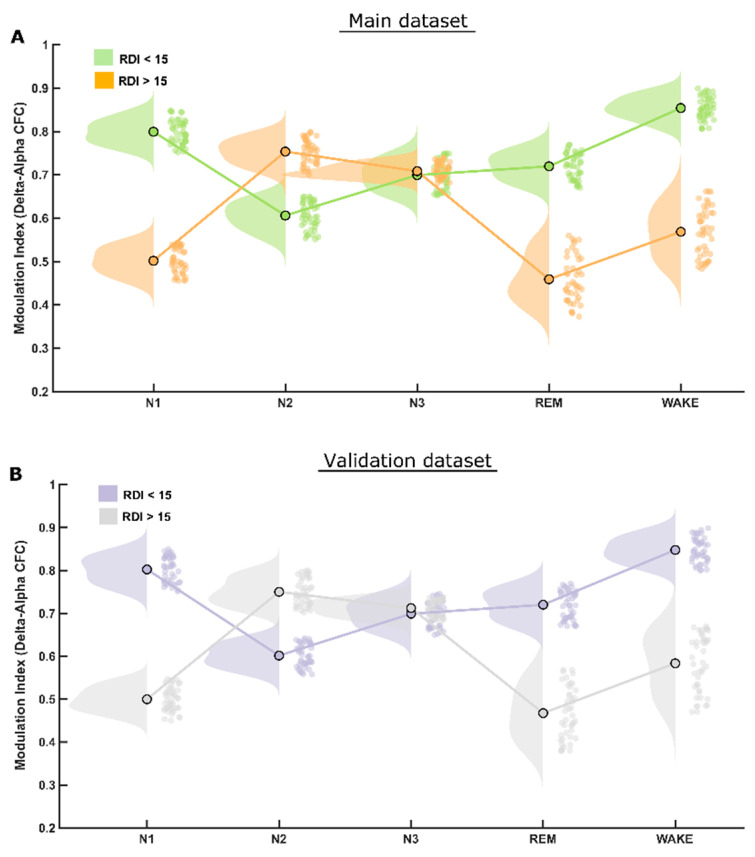
*MI differences in delta–alpha CFC.* (**A**) The raincloud plot illustrates that patients of the RDI > 15/h group have significantly lower delta–alpha CFC modulation index than those in REM and N1 sleep stages and significantly higher MI in N2 sleep stage than that of patients in the RDI ≤ 15/h group. The delta–alpha CFC modulation index in NREM-3 (N3) sleep stage is almost identical in the two patient groups. (**B**) Of note, exactly the same pattern was found again in both the initial and the validation datasets.

However, the CFC-MI at the delta–alpha frequency bands was reduced significantly (*p* < 0.001) only during REM and N1, but not in the N3 sleep stage in patients with clinically significant OSA when compared to patients with mild or no OSA (RDI ≤ 15/h), as depicted in Figure 3A. Furthermore, the CFC-MI was higher in the NREM sleep stage N2 in patients with significant OSA (i.e., RDI > 15/h) than in those without (RDI ≤ 15/h). Please refer to Supplementary Table S1 for all the values.

**Figure 4:**
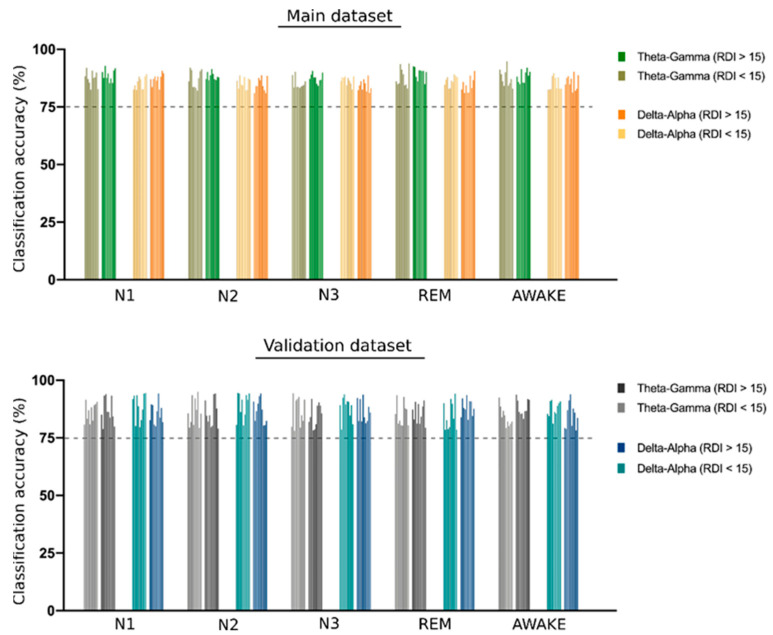
*SVM classification of different sleep stages*. The bar plot shows the support vector machine (SVM) classification accuracy of different sleep stages using the theta–gamma and delta–alpha cross frequency coupling (CFC) modulation indices. A group of 10 bars in each set represents the accuracy obtained for 10-fold cross-validation. A dotted line is shown at 75% accuracy to emphasize the significant degree of classification obtained for all CFC metrics used in the study. All the accuracy values are presented in the Supplementary Table S2.

The support vector machine (SVM) analysis showed a significant classification of all four sleep stages and of the awake stage using CFC modulation indices separately from both the theta–gamma and delta–alpha frequency bands. All classification accuracy was higher than 80%, reaching up to 94% for the classification of awake stage using theta–gamma CFC-MI as shown in Figure 4.

**Figure 5:**
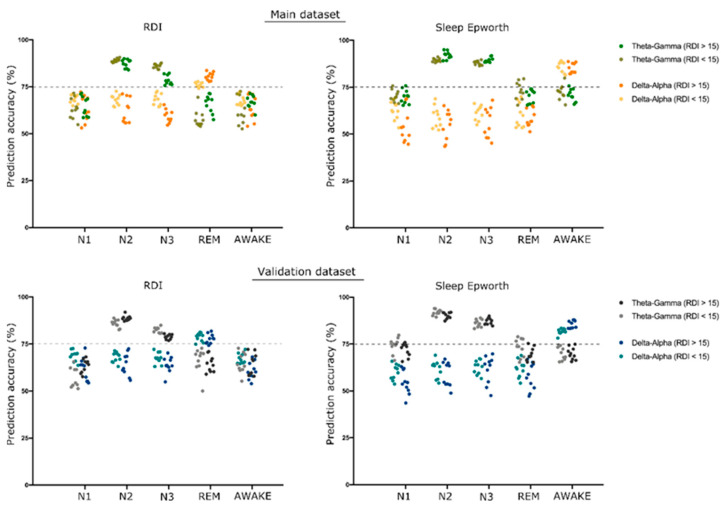
*SVM prediction of RDI and ESS*. The scatterplot shows the support vector machine (SVM) prediction accuracy of respiratory distress index (RDI) and Epworth Sleepiness Scale (ESS) using theta–gamma and delta–alpha cross-frequency coupling (CFC) modulation indices. A group of 10 dots in each set represents the accuracy obtained for 10-fold cross-validation. We considered accuracy above 75% to be significant, represented by a dotted line on the graph.

In addition, we were able to predict, using SVM, the RDI and the Epworth Sleepiness Scale (ESS) in different sleep stages with significant accuracy (more than 75%) using the CFC-MI in both couples of frequency bands. The theta–gamma CFC was able to significantly predict the RDI and ESS in NREM (N2 and N3) sleep stages. The delta–alpha CFC in the REM sleep stage was able to significantly predict RDI, and the delta–alpha CFC in awake stages was able to significantly predict ESS. The details of all predictions are shown in Figure 5.

### 3.2. Validation Dataset

Among the 84 patients analyzed from this dataset, 42 patients were diagnosed with a respiratory distress index (RDI) ≤ 15 per hour, and 42 patients were diagnosed with clinically significant OSA (3 patients with RDI between 15 and 30 per hour, and 39 patients with RDI > 30 per hour). For this dataset as well, the two groups did not significantly differ in terms of age and sex (*p* > 0.05). The demographic details are presented in Table 1.

The statistical analysis performed for this dataset yielded quite similar results to that of the first (main) dataset, therefore validating most of the findings. The CFC-MI at the theta–gamma frequency bands were also reduced for clinically significant OSA patients (RDI > 15/h), similar to those in the main findings, also showing higher modulation during NREM-N2 and N3 sleep stages (Figure 2B). Likewise, CFC-MI at the delta–alpha frequency bands was also reduced significantly only during REM and N1 but not in N2 and N3 sleep stages in patients with clinically significant OSA, as found in the main dataset (Figure 3B).

The SVM analysis for classifying sleep stages using CFC modulation indices for both the theta–gamma and delta–alpha frequency bands showed replicable results using the validation dataset with all classification accuracy above 80%, as shown in Figure 4.

Similarly, the validation dataset was further able to replicate the prediction results for clinical parameters—RDI and Epworth Sleepiness Score (ESS)—with accuracy higher than 75% using the same CFC modulation indices as in the main dataset, as shown in Figure 5.

**Figure 6:**
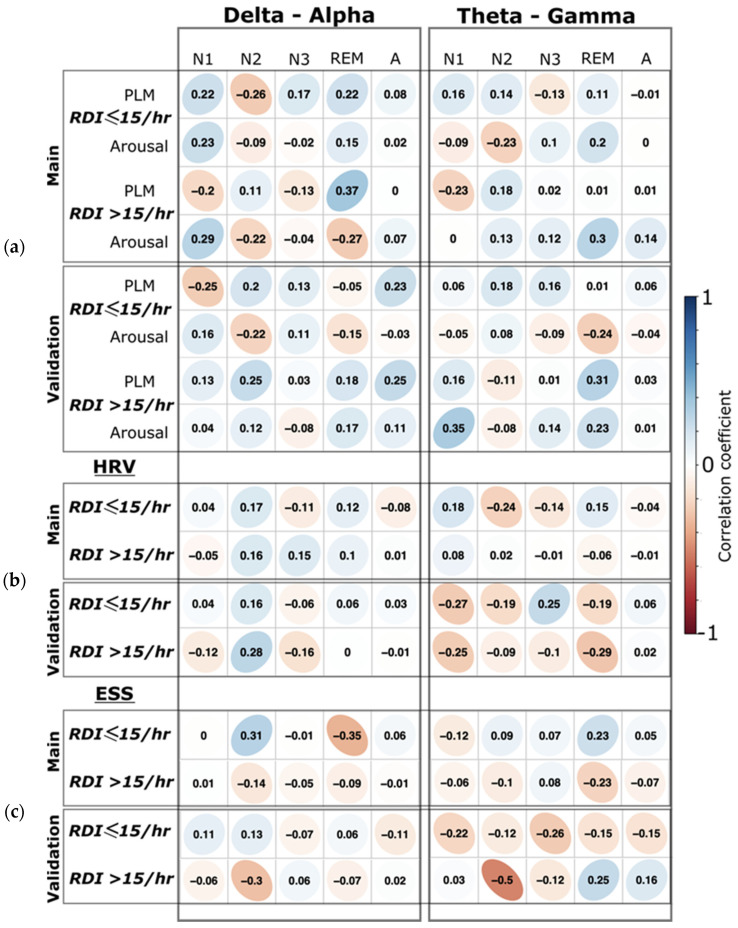
Correlation of clinical parameters with CFC measures. (**a**) The correlation coefficients between arousal and periodic leg movement indices (PLM) to the phase–amplitude cross-frequency coupling (PACFC) for each sleep stage separately. For both delta–alpha and theta–gamma, PACFC are separated by the columns. (**b**) The correlation coefficients between the heart rate variability (HRV) and the PACFC in the different sleep stages are separated by columns. (**c**) The correlation coefficients between Epworth Sleepiness Score (ESS) and the PACFC in different sleep stages. Correlations for the main and the validation groups are shown in each row separately. The r values are shown for correlation. All correlations were non-significant (*p* > 0.05).

However, the correlation between the Epworth Sleepiness Score (ESS) and PACFC was also not significant in any sleep stage (Figure 6). All the confusion matrices from the SVM analyses are presented in Supplementary Table S3.

### 3.3. Scientific Control Findings

**Figure 7:**
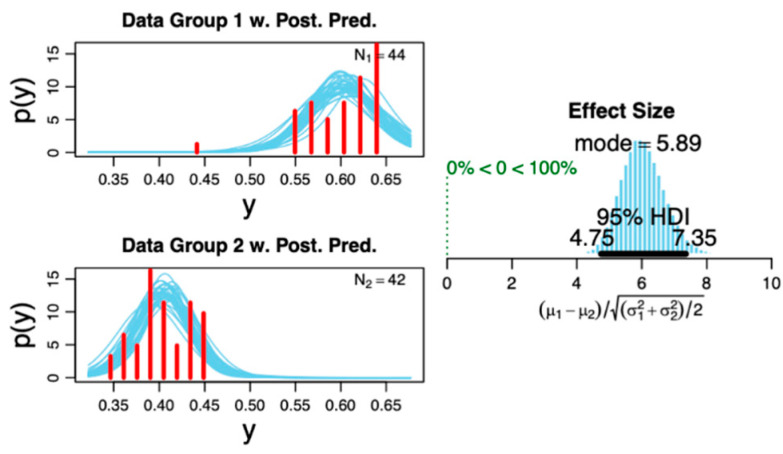
Posterior distribution of the studied groups. The plot on the right shows the distribution histogram of the effect size displaying the 95% high-density interval (HDI), which is within the analyzed data. This indicates a sufficient sample size of included subjects based on the primary outcome (i.e., phase–amplitude cross-frequency coupling for theta–gamma at the N1 sleep stage). The plots on the left show the probability distribution with superimposed posterior predictive distribution of raw data for each data sample. Please refer to Ref [43] for methodological details regarding the distributions.

(1) We did not find any significant correlation (all *p* > 0.05) between the arousal and PLM indices to the PACFC at any sleep stage (Figure 6). (2) Moreover, we did not find significant correlation between the heart rate variability and PACFC at any sleep stage, showing no influence of the autonomic nervous system on PACFC (Figure 6). (3) The Bayesian posterior distribution [43] showed that the 95% high-density interval (HDI) is within the obtained effect in our analyzed data (Figure 7), indicating a sufficient sample size for the primary outcome in this study.

## 4. Discussion

We found a significant reduction in the theta–gamma modulation index (MI) at the central sensorimotor cortical regions in patients with moderate or severe OSA compared to patients with mild OSA or healthy individuals. The MI reduction during sleep was frequency-band specific; it involved the theta–gamma connectivity during all sleep stages while involving delta–alpha connectivity only during REM and N1. Therefore, a global reduction in modulation (both theta–gamma and delta–alpha) was observed during REM and N1. Moreover, the MI differences among the stages were so prominent that, in both datasets, sleep stage classification using MI values was achieved in both patient groups. Additionally, theta–gamma MI during N2 and N3 very reliably predicted both RDI and ESS, and delta–alpha MI during REM very reliably predicted RDI.

These novel findings showing the functional disconnection between theta and gamma activity in the cortical sensorimotor area throughout all sleep stages in OSA patients have pathophysiological and clinical implications that need to be further discussed.

### 4.1. Theta–Gamma Phase–Amplitude Coupling

Theta–gamma PACFC has been linked to motor, sensory and cognitive processes [10,11]. In individuals with RDI ≤ 15/h, a very strong coupling between theta and gamma oscillations exists in both N2 and N3 stages. In moderate and severe OSA, the modulation index decreases quite significantly during N3 while at a much lesser extent in N2. During short periods of wakefulness between the sleep stages, the MI increased significantly in patients with moderate/severe (i.e., significant) OSA compared to those with RDI ≤ 15/h. This may reflect a physiological (motor, respiratory, cognitive) compensatory role for cortical arousals and/or intermittent wakefulness periods to promote transient increases in central sensorimotor connectivity in patients with significant OSA. A net synaptic potentiation associated with wakefulness states [44] may provide the ground for such an increase in connectivity.

The recorded sensorimotor cortex neural activity may either be primarily generated at this anatomical area or may be an epiphenomenon of the activity of other subcortical/thalamic or brainstem neural master generators. These generators promote neuromodulation of the cranial nerve pathways, causing the reduced upper airway muscular tone associated with the upper airway obstructions found during respiratory events in sleep in OSA [5,45,46,47].

In patients with focal epileptic seizures, theta–gamma phase–amplitude coupling intensity during sleep was the highest during N3 and the lowest during REM [48]. In our moderately and severely affected OSA patients, theta–gamma PACFC was the highest during N2 (Figure 2). In agreement with the Amiri et al. findings, the coupling of fast to slow oscillations was much more reduced during REM compared to N2 and N3 in all OSA patient groups; this reduction was more prominent in patients with significant OSA. Strong CFC between high-frequency and slow-wave oscillations during slow-wave sleep was found in anesthetized primates’ hippocampus [49]. EEG measures the summed postsynaptic potentials of synchronously active regions of the cortex and hippocampus that has spread through the brain, skull and scalp. Although they often coincide, it is not necessary that the firing of action potentials is related to oscillations of postsynaptic potentials. Hence, the oscillations of postsynaptic potentials do not always result in the firing of postsynaptic action potentials [50]. It is difficult to distinguish cortical from hippocampal output in humans based solely on surface EEG recordings.

### 4.2. Delta–Alpha Phase–Amplitude Coupling

A significant increase in delta–alpha CFC-MI is observed during N2 in patients with significant OSA compared to patients with RDI ≤ 15/h. This finding may represent a compensatory increased activity of the sensorimotor cortex during the respiratory-event-rich N2 stage to exert a better motor control of respiration in more severely (RDI > 15/h) affected OSA patients.

Delta band oscillation in spike and local field potentials activity in the somatosensory whisker barrel cortex of awake mice is phase locked to respiration [51]. Therefore, respiratory activity directly modulates slow (1–4 Hz) rhythmic neuronal activity in the somatosensory whisker barrel cortex and indirectly modulates gamma band power through phase–amplitude coupling mechanisms in mice. Our findings provide preliminary evidence of a physiological involvement of delta and gamma band oscillations in the control of respiration in humans, especially in OSA, and should be further validated.

Notably, the delta–alpha CFC MI remains quite stable during N3, regardless of OSA severity. Therefore, delta–alpha coupling may be implicated in brain connectivity processes that remain stable during N3, or it may also be a surrogate marker of the well-known respiratory stability during N3, when apneas and hypopneas occur much less frequently.

### 4.3. Sleep Stage Classification

Given that both theta–gamma and delta–alpha modulation indices can reliably predict sleep stage classification according to AASM criteria, it can be suggested that quite distinct sleep-stage-specific PACFC patterns involving the aforementioned frequency bands exist. These distinct patterns are apparently quite robust, involve at least the two aforementioned (gamma–theta, delta–alpha) oscillatory channels and may involve further oscillatory channels. Moreover, given that a large proportion of the tested datasets belong to patients with RDI > 15/h, these sleep-stage-specific coupling patterns appear to remain quite robust, irrespective of the degree of concomitant sleep-related respiratory distress.

### 4.4. REM Sleep

The global (theta–gamma and delta–alpha) connectivity reduction, as depicted by the MI reduction, during REM at the central sensorimotor areas in OSA patients with RDI > 15/h compared to patients with RDI ≤ 15/h may provide a surrogate marker of the reduced central motor output during REM. This reduced motor output probably involves many muscular groups and, in particular, involves the muscles controlling the upper airway patency, given its strong correlation with the RDI. The MI difference is particularly prominent in the case of delta–alpha CFC-MI (Figure 2). A differential modulation of global and local oscillations during REM sleep has been reported [52]. Therefore, the multi-frequency (global) MI at the sensorimotor areas during REM may serve as a surrogate marker of OSA disease severity. In support of this argument, a further analysis (Figure 4) shows that delta–alpha MI during REM very reliably predicted average RDI in both tested datasets.

### 4.5. Patient-Reported Excessive Daytime Sleepiness

Theta–gamma MI during N2 and N3 and delta–alpha MI during short periods of wakefulness from sleep emerge as reliable surrogate markers of patient-reported excessive daytime sleepiness (EDS). These findings also point to possible sleep-related oscillation and stage-specific physiological mechanisms that promote attention and vigilance in humans. Subjective ratings of sleepiness have exhibited pronounced associations with increased functional connectivity in widespread regions within the sensorimotor network [15].

### 4.6. Cross-Frequency Coupling and Brain Function

Phase–amplitude cross frequency coupling (PACFC) modulation index was the primary outcome measure and was tested as a predictor of the clinical variables in this study. Hence, we feel that a brief summary of its role in brain function is necessary for readers’ convenience. PACFC and its modulation has been reported for different tasks, implicating its association with the underlying physiology. Spatial working memory performance [53], multi-item working memory maintenance [54], perceptual outcomes alterations [55], learning [56], visual attention and perception [57] are some of the functional characteristics that have been associated with PACFC modulation. Moreover, it has also been shown to contribute to the BOLD (blood–oxygen level-dependent) connectivity [58] and to have associations with brain alterations found in several neurological disorders, including epilepsy [59], Parkinson’s disease [60,61], Alzheimer’s disease [62], schizophrenia [12], obsessive compulsive disorder (OCD) [63] and mild cognitive impairment (MCI) [64]. Even though there is enough evidence of PACFC being potentially a promising approach to unravel brain function and some of its pathologies with a credible physiological mechanism (low-frequency phase reflects local neuronal excitability, while high-frequency power increases reflect either a general increase in population synaptic activity or the selective activation of a connected neuronal subnetwork), there are still several unanswered questions regarding the origination, causation and mechanism of these oscillations [10,65]. The choice of modulation index (MI) in this study is based on the finding that, among some of the most widely used phase–amplitude coupling measures, MI has been shown to be most robust against confounding influences of moderators, including data length, signal-to-noise ratio and sampling rate, when approaching the Nyquist frequencies [33].

### 4.7. Therapeutic and Clinical Practice Implications

The aforementioned evidence may open new possibilities of intervention through pharmacological or transcranial magnetic stimulation (TMS) of the sensorimotor cortex in OSA patients [66]. TMS during sleep has been applied on corticomotor-somatotopic representation of the tongue; induced twitches have briefly improved airflow without causing arousals in OSA patients [67]. However, the effect on other motor areas and the neurocognitive effect of TMS has not been extensively studied in OSA. The finding that the aforementioned CFC modulation indices at both frequency bands could significantly predict the RDI and Epworth Sleepiness Score (ESS) in OSA patients should be further validated in larger studies.

Our findings that (1) theta–gamma CFC-MI significantly predicted the RDI and ESS in NREM (N2, N3), (2) delta–alpha CFC-MI in REM predicted RDI, and (3) delta–alpha CFC-MI in awake states significantly predicted ESS, suggest that the CFC-MI theta–gamma and delta–alpha metrics may depict quite different processes in human sleep physiology. Delta–alpha coupling appears to be significant (1) for the control of motor upper airway stability and respiration during REM sleep and (2) for the control of attention and alertness processes, as depicted by ESS, during (cortical) arousal and short periods of wakefulness between sleep stages. Theta–gamma phase–amplitude coupling appears to be very significant (1) for the control of upper airway stability and respiration during NREM N2 and N3 sleep and (2) for attention- and alertness-related processes (depicted by ESS) that occur during the N2 and N3 sleep. These findings suggest that the cortical central sensorimotor regions may indeed be a significant hub in the networks regulating sleep and/or sleep-related breathing activity. After validation in larger cohorts of patients, the modulation index could be eventually incorporated as an additional metric depicting both the severity of respiratory distress as well as the daytime sleepiness in patients with OSA.

The replication of the findings in the validation dataset further supports the reproducibility and validity of the results and points to their clinical significance as diagnostic surrogate markers. Moreover, the scientific control findings clearly evidenced no influence of arousal, periodic leg movement, as well as that of the autonomic nervous system in the PACFC measure.

### 4.8. Implications for Future Research and Limitations

We propose that the theta–gamma MI at the sensorimotor cortical areas during N2 and N3, and the delta–alpha CFC-MI at the sensorimotor cortical areas during REM, may be used as metrics of respiratory distress during sleep in humans and, hence, metrics of OSA severity. Therefore, further studies of theta–gamma CFC during N2 and N3 and delta–alpha CFC during REM should be pursued. Computing these MIs at further cortical areas may provide additional insights in OSA pathogenesis and diagnosis. Neurophysiologic and neuroimaging studies of thalamo-cortical connectivity based on the present findings may further elucidate the mechanisms of excessive daytime sleepiness [68,69]. Furthermore, it would be interesting to assess the effect of established evidence-based therapies for OSA, such as positive airway pressure (PAP) therapy, on PACFC.

There are a few limitations of the study. All participants in the study were individuals who were present with complaints of snoring, daytime sleepiness or other sleep-disordered breathing (SDB) issues. Hence, a control group with healthy individuals without SDB or OSA-related complaints were missing. We excluded all patients with known neurological or psychiatric comorbidities from the study. However, patients with less clinically significant comorbidities, such as diabetes mellitus, arterial hypertension and the associated medication, were not excluded. Although not up to a significant level, these factors might have confounded some findings of this study.

## 5. Conclusions

Central cortical sensorimotor area functional disconnection between theta and gamma activity is observed throughout all sleep stages in OSA. An additional significant delta–alpha sensorimotor area disconnection occurs during the REM and N1 stages in OSA. Hence, sensorimotor disconnection is highly prevalent, shows frequency-band and sleep-stage-specific patterns and further provides evidence of the existence of a central sensorimotor dysfunction in OSA patients. Theta–gamma modulation index during N2 and N3 reliably predicted patient-reported sleepiness. Therefore, modulation indices may be used as surrogate diagnostic predictive markers of respiratory distress during sleep and of patient-reported excessive daytime sleepiness.

## Figures and Tables

**Table 1:** Demographic details of all the participants included in the study. Here, RDI: respiratory disturbance index; ESS: Epworth Sleepiness Scale.

Dataset	Group	N	Age (Years)	Sex	RDI (per Hour)	ESS	*t*-Test *p*-Values
Main	RDI ≤ 15	42	55.67 ± 10.22	F = 19	9.30 ± 3.26	10.90 ± 4.53	Age: 0.746
				M = 23			Sex: 0.085
	RDI > 15	44	56.52 ± 13.91	F = 12	27.96 ± 12.47	11.59 ± 4.45	RDI: <0.001
				M = 32			ESS: 0.480
Validation	RDI ≤ 15	42	52.79 ± 9.71	F = 17	11.04 ± 2.97	9.86 ± 4.90	Age: 0.119
				M = 25			Sex: 0.168
	RDI > 15	42	56.0 ± 9.02	F = 11	49.48 ± 19.67	10.14 ± 5.47	RDI: <0.001
				M = 31			ESS: 0.817

## Data Availability

The data used in the manuscript may be made available in anonymized form to research institutes, given an appropriate ethics and research approval.

## Supplementary Materials

The following supporting information can be downloaded at: https://www.mdpi.com/article/10.3390/biology11050700/s1, Table S1: Mean, standard deviation and *p*-values (*t*-test) of Modulation indices as presented in result section; Table S2: All CFC-modulation index values as discussed in result section; Table S3: Normalized confusion matrices for the SVM analysis.
