# Supplementary material for: Reduced Cross-Frequency Coupling and Daytime Sleepiness in Obstructive Sleep Apnea Patients
Source: Biology (Basel). 2022 May 2;11(5):700. doi: 10.3390/biology11050700 (PMC9138271; doi:10.3390/biology11050700)
Supplement: Supplementary file 1 [file biology-11-00700-s001.zip › biology-1639525-supplementary.pdf]

Supplementary Table S1: Mean, standard deviation and p-values (t-test) of Modulation indices as presented in result section

Theta-Gamma						
Sleep stages	Main			Validation		
	RDI < 15	RDI > 15	**t-test p-values**	RDI < 15	RDI > 15	**t-test p-values**
N1	0.63 ± 0.03	0.43 ± 0.03	1.47E-46	0.60 ± 0.03	0.41 ± 0.03	2.14E-44
N2	0.75 ± 0.03	0.73 ± 0.03	3.32E-06	0.75 ± 0.03	0.70 ± 0.03	4.54E-06
N3	0.78 ± 0.03	0.56 ± 0.03	2.29E-36	0.75 ± 0.02	0.60 ± 0.03	2.27E-35
REM	0.57 ± 0.03	0.47 ± 0.06	3.20E-13	0.56 ± 0.03	0.44 ± 0.05	1.26E-15
Wake	0.52 ± 0.03	0.82 ± 0.03	1.08E-57	0.54 ± 0.03	0.82 ± 0.03	2.64E-56
Delta-Alpha						
Sleep stages	Main			Validation		
	RDI < 15	RDI > 15	**t-test p-values**	RDI < 15	RDI > 15	**t-test p-values**
N1	0.82 ± 0.03	0.48 ± 0.03	1.72E-58	0.77 ± 0.03	0.54 ± 0.03	1.18E-59
N2	0.60 ± 0.03	0.78 ± 0.03	1.05E-33	0.59 ± 0.03	0.77 ± 0.03	5.13E-36
N3	0.70 ± 0.03	0.70 ± 0.02	0.068910504	0.74 ± 0.03	0.72 ± 0.02	0.051000447
REM	0.73 ± 0.03	0.51 ± 0.05	1.58E-39	0.68 ± 0.03	0.39 ± 0.06	9.70E-36
Wake	0.88 ± 0.03	0.55 ± 0.06	1.91E-42	0.89 ± 0.03	0.60 ± 0.07	1.38E-35

Supplementary Table S2: All CFC-modulation index values as discussed in result section.

Sleep Stages	10 fold Cross-Validation	Main dataset (Accuracy)				Validation dataset (Accuracy)			
		Delta-Alpha (RDI < 15)	Delta-Alpha (RDI > 15)	Theta-Gamma (RDI < 15)	Theta-Gamma (RDI > 15)	Delta-Alpha (RDI < 15)	Delta-Alpha (RDI > 15)	Theta-Gamma (RDI < 15)	Theta-Gamma (RDI > 15)
**N1**	1	82.49636177	87.0478972	88.33884908	90.1568646	91.85030267	82.6924263	80.75709924	85.09354017
	2	84.38595178	83.6169564	91.99645743	87.62149499	93.39846293	89.5549455	91.50283719	78.84412532
	3	82.37081012	87.1919322	87.14808769	92.84551681	80.15877588	89.1366661	83.29065571	93.34617387
	4	86.04342514	88.2404461	85.54820432	87.61965587	93.52738955	80.7643995	86.9850633	94.06138223
	5	88.09140709	86.415043	82.48405024	89.58604752	88.75010719	80.0229606	80.8160284	86.34468957
	6	87.04855999	88.1403556	90.75283388	85.38932825	79.65818688	86.4721889	88.233693	86.31729485
	7	82.71913321	82.5830961	87.58433797	87.43210684	82.73446972	94.3156473	82.47051184	83.74122997
	8	82.64689939	88.084533	87.87261005	85.45020575	87.29698583	83.7865574	89.11934467	93.30091539
	9	88.21792429	90.6765838	89.9281432	90.82301188	94.2776162	87.9495518	89.71664655	84.27719528
	10	89.24107795	89.5362332	82.77224286	91.8400001	94.4031051	81.804803	90.71857708	79.89044684
**N2**	1	86.27017561	80.9485798	86.1055487	87.1569907	80.67942239	90.77154	85.65920717	91.26428516
	2	82.8732337	84.0308028	92.0453877	90.16982138	94.50007729	82.336617	79.42496343	84.62556023
	3	88.60647086	84.0611868	91.05362886	86.6473377	94.27183812	86.6012699	81.89260847	82.10875186
	4	84.70478175	87.5353132	83.62931835	88.77566373	86.25138603	89.8843043	93.52673515	84.86650647
	5	84.35178442	86.577358	83.69196886	91.49181048	91.60476797	93.1453553	80.59042632	79.63972693
	6	87.97050742	88.6830034	83.2005804	89.37741173	80.41206776	94.3079542	92.03888862	80.24354597
	7	82.08269295	84.0441821	82.10166382	87.01780198	85.1699418	87.302664	87.1518214	94.01486004
	8	82.38757871	82.2663065	87.50042201	86.69302521	93.56750393	80.3566155	94.93429018	94.25428718
	9	87.34332897	80.953332	90.52245127	88.07333287	91.4675246	80.5379981	79.32898399	87.77854612
	10	86.82774387	88.4912731	91.39799282	87.80381088	94.31137125	82.3776403	85.52553059	79.01625223
**N3**	1	86.20881973	82.2624197	88.90572082	87.23645686	89.14759189	92.2921934	79.81309709	81.99125853
	2	87.83767559	84.2709879	83.4146332	89.0248728	78.60709854	82.322797	94.35226737	84.00369571
	3	87.65802788	86.0695639	90.21296286	90.68290829	92.4351982	91.842842	78.07878181	91.96029868
	4	88.25101641	82.5184858	83.64449825	87.81901305	93.87788521	82.1399245	91.1734779	78.26185844
	5	84.3038158	87.0613468	83.74594296	87.8642021	89.53849763	93.7974816	91.89415475	78.73140463
	6	87.54025589	85.3292841	83.28172321	85.12385483	90.88158222	83.949724	92.76780999	80.8728305
	7	86.45335868	81.6703008	83.84635423	84.21990614	90.63325196	81.3421193	79.43540937	89.03496307
	8	85.17216634	88.6012516	84.18726688	86.61166739	84.66785933	82.2684256	84.79630503	90.43928056
	9	82.49272534	81.1066695	84.55123599	86.85768525	89.14312413	88.4727595	82.41779685	89.01168137
	10	88.24140425	83.2347297	86.12723708	89.88321121	80.91017369	86.0459104	91.60116416	85.66570301
**REM**	1	84.70067091	82.6111032	86.11357699	92.61242332	90.0027835	83.9782116	85.33403507	87.29915117
	2	86.86292726	85.8395948	84.82832302	92.42157786	78.54115839	92.1240867	93.48100911	83.0374537
	3	87.9300324	81.006486	85.263544	88.120977	82.70769074	87.9494895	81.09139948	90.65977772
	4	82.83850594	84.4584696	93.60799127	86.16430557	78.78491364	87.3453013	82.48464958	81.21223526
	5	83.02310704	81.1533087	91.14190192	90.8750815	79.65124028	93.5922923	80.47416267	89.67518237
	6	86.39632086	81.2351236	89.22459326	90.83394645	91.99878308	82.8592633	80.3131655	81.11968965
	7	85.88183527	88.6287068	84.39763768	90.66583258	89.81208659	90.8724039	92.77796753	84.26423814
	8	89.12380543	83.2072735	84.75640095	90.69319507	83.39069116	90.8133946	87.85497799	88.63551553
	9	88.39168223	86.6388678	83.00550851	84.95328375	94.15377483	84.4675794	87.34762343	91.2638664
	10	87.87472867	90.6086493	93.87940534	90.13404387	78.58558337	87.6529679	80.46423157	79.37913807
**AWAKE**	1	82.41065509	84.7573349	91.18729783	88.16934521	85.45865411	79.2895229	92.501529	93.79956151
	2	82.58308239	87.6422741	89.25125657	85.90946885	84.48649377	78.917152	88.57493724	91.18711554
	3	82.70821968	88.339092	86.07457687	84.88666864	91.0137854	87.0235584	83.96619048	86.27545775
	4	88.38680691	84.7590656	84.16064632	91.41217027	91.51839832	91.2458429	86.72524218	85.40959601
	5	89.54406512	87.2104784	90.09246437	85.57508764	81.17683428	93.8781816	84.83073657	85.59532374
	6	87.46972458	81.2073056	94.84315155	85.47212919	86.32599473	80.2084055	79.29143376	83.20794102
	7	83.05666365	90.2713583	84.2156163	89.99388495	85.57496541	87.6700022	82.07857461	86.64464714
	8	87.78179632	82.0620689	85.35129926	92.04950438	88.98732117	85.9796409	80.09642189	86.68311659
	9	82.88282785	82.9279672	87.15839114	88.64902388	90.05920212	78.2023352	81.1264324	91.89967104
	10	82.93994282	88.7761329	82.961932	90.32432076	90.82967359	83.731085	82.07919294	91.51213409

Supplementary Table S3: Normalized confusion matrices for the SVM analysis

Normalized Confusion Matrices for the SVM analysis

Main Group - Delta Alpha

RDI ≤ 15/h

	Predicted values					
Actual Values	Sleep stages	N1	N2	N3	REM	WAKE
	N1	0.86	0.10	0.03	0.01	0.00
	N2	0.06	0.82	0.10	0.02	0.00
	N3	0.03	0.12	0.83	0.02	0.00
	REM	0.00	0.02	0.10	0.84	0.04
	WAKE	0.00	0.00	0.04	0.09	0.87

RDI > 15/h

	Predicted values					
Actual Values	Sleep stages	N1	N2	N3	REM	WAKE
	N1	0.83	0.11	0.04	0.02	0.00
	N2	0.12	0.82	0.05	0.01	0.00
	N3	0.05	0.12	0.81	0.02	0.00
	REM	0.00	0.03	0.08	0.84	0.05
	WAKE	0.00	0.00	0.03	0.08	0.89

Main Group - Theta Gamma

RDI ≤ 15/h

	Predicted values					
Actual Values	Sleep stages	N1	N2	N3	REM	WAKE
	N1	0.90	0.08	0.02	0.00	0.00
	N2	0.10	0.84	0.06	0.00	0.00
	N3	0.02	0.13	0.83	0.02	0.00
	REM	0.00	0.00	0.10	0.85	0.05
	WAKE	0.00	0.00	0.01	0.05	0.94

RDI > 15/h

	Predicted values					
Actual Values	Sleep stages	N1	N2	N3	REM	WAKE
	N1	0.90	0.08	0.02	0.00	0.00
	N2	0.10	0.85	0.05	0.00	0.00
	N3	0.00	0.11	0.86	0.03	0.00
	REM	0.00	0.01	0.09	0.86	0.04
	WAKE	0.00	0.00	0.02	0.06	0.92

Validation Group - Delta Alpha

RDI ≤ 15/h

	Predicted values					
Actual Values	Sleep stages	N1	N2	N3	REM	WAKE
	N1	0.84	0.11	0.05	0.00	0.00
	N2	0.12	0.80	0.08	0.00	0.00
	N3	0.02	0.11	0.87	0.00	0.00
	REM	0.00	0.00	0.10	0.88	0.02
	WAKE	0.00	0.00	0.02	0.08	0.90

RDI > 15/h

	Predicted values					
Actual Values	Sleep stages	N1	N2	N3	REM	WAKE
	N1	0.82	0.13	0.04	0.01	0.00
	N2	0.13	0.81	0.06	0.00	0.00
	N3	0.01	0.08	0.89	0.02	0.00
	REM	0.00	0.01	0.09	0.86	0.04
	WAKE	0.00	0.00	0.03	0.09	0.88

Validation Group - Theta Gamma

RDI ≤ 15/h

	Predicted values					
Actual Values	Sleep stages	N1	N2	N3	REM	WAKE
	N1	0.88	0.07	0.04	0.01	0.00
	N2	0.09	0.86	0.05	0.00	0.00
	N3	0.02	0.14	0.81	0.03	0.00
	REM	0.00	0.01	0.11	0.83	0.05
	WAKE	0.00	0.00	0.02	0.06	0.92

RDI > 15/h

	Predicted values					
Actual Values	Sleep stages	N1	N2	N3	REM	WAKE
	N1	0.89	0.07	0.04	0.00	0.00
	N2	0.10	0.86	0.04	0.00	0.00
	N3	0.00	0.11	0.85	0.04	0.00
	REM	0.00	0.01	0.10	0.84	0.05
	WAKE	0.00	0.00	0.02	0.08	0.90
